# Exploring the most stable aptamer/target molecule complex by the stochastic tunnelling-basin hopping-discrete molecular dynamics method

**DOI:** 10.1038/s41598-021-90907-y

**Published:** 2021-06-01

**Authors:** Chia-Hao Su, Hui-Lung Chen, Shin-Pon Ju, Tai-Ding You, Yu-Sheng Lin, Ta-Feng Tseng

**Affiliations:** 1 Institute for Translational Research in Biomedicine, Kaohsiung Chang Gung Memorial Hospital, Kaohsiung, 833 Taiwan, ROC; 2 Department of Chemistry and Institute of Applied Chemistry, Chinese Culture University, Taipei, 111 Taiwan, ROC; 3 Department of Mechanical and Electro-Mechanical Engineering, National Sun Yat-Sen University, Kaohsiung, 80424 Taiwan, ROC; 4 Department of Medicinal and Applied Chemistry, Kaohsiung Medical University, Kaohsiung, 80708 Taiwan, ROC

**Keywords:** Computational biology and bioinformatics, Structural biology

## Abstract

The stochastic tunnelling-basin hopping-discrete molecular dynamics (STUN-BH-DMD) method was applied to the search for the most stable biomolecular complexes in water by using the MARTINI coarse-grained (CG) model. The epithelial cell adhesion molecule (EpCAM, PDB code: 4MZV) was used as an EpCAM adaptor for an EpA (Apt_EpA_) benchmark target molecule. The effects of two adsorption positions on the EpCAM were analysed, and it is found that the Apt_EpA_ adsorption configuration located within the EpCAM pocket-like structure is more stable and the energy barrier is lower due to the interaction with water. By the root mean square deviation (RMSD), the configuration of EpCAM in water is more conservative when the Apt_EpA_ binds to EpCAM by attaching to the pocket space of the EpCAM dimer. For Apt_EpA_, the root mean square fluctuation (RMSF) analysis result indicates Nucleobase 1 and Nucleobase 2 display higher flexibility during the CGMD simulation. Finally, from the binding energy contour maps and histogram plots of EpCAM and each Apt_EpA_ nucleobase, it is clear that the binding energy adsorbed to the pocket-like structure is more continuous than that energy not adsorbed to the pocket-like structure. This study has proposed a new numerical process for applying the STUN-BH-DMD with the CG model, which can reduce computational details and directly find a more stable Apt_EpA_/EpCAM complex in water.

## Introduction

With the continuous progress in new drug and medical developments, some incurable diseases in the past have become curable after patients have found an effective treatment^1–3^. However, the global death toll for cancer or cancer-related diseases still displays an increasing trend year after year. The main reason causing cancer death is diagnosis at later stages. If the cancer cells first appearing in the human body can be precisely identified, opportunities to prevent the proliferation of cancer cells by proper treatment are substantially increased. Consequently, a method which can precisely identify specific cancer cells at early stages is a key technology for cancer treatment. Currently, these methods are primarily rapid screening^4,5^, imaging^6,7^, and genetic testing^8,9^. However, not all cancer cells can be found at the initial stage or when cancer cells are scarce because of the limitation of these methods.

Among the methods for recognizing the specific cancer cells, single-stranded deoxyribonucleic acid (ssDNA) aptamers with a specific nucleobase sequence can be utilized to identify the particular protein target molecule on a specific cancer cell. One can further confirm the existence of such cancer cells once the ssDNA aptamer recognizes the targeted protein. Most aptamer molecules are ssDNA and ribonucleic acid (RNA) because their unique molecular structures display a shape complementary to the targeted molecules, resulting in a higher binding strength and higher affinity between the aptamer molecules and the target molecules. Consequently, using the aptamer for target molecule recognition has been widely applied in the field of biotechnology. For example, in Wang’s study^10^, the tumor DNA aptamer was combined with porous silicon nanoparticles stuffed by drug molecules. After the tumor DNA aptamer attached to the tumor cell, drugs within the silicon nanoparticles could be precisely applied to the tumor cells. This method can significantly decrease the drug’s side effects and improve its efficiency. In Irshad's study^11^, their experimental results show the aptamers not only can be used to identify the target molecules, but can also be used to inactivate the target molecules by means of the aptamer binding to the target molecules. He’s study^12^ used two aptamers (HF3-58 and HA5-68) which were selected by systematic evolution of ligands by exponential enrichment (SELEX), and which were shown to inhibit paclitaxel (A2780T) from ovarian cancer. It has been confirmed that these two aptamers have the good characteristics of a stable structure and drug-resistance in detecting ovarian cancer. Although SELEX is a well-developed technology to obtain an optimal aptamer sequence with high specificity and affinity to a specific target protein, the high cost and long time required for the SELEX filtering process still require improvement^13^.

In addition to the high cost and the time of SELEX process, the interaction mechanism between the aptamer and the target molecule is also very difficult to investigate using an experimental approach. A molecular simulation with a biomolecular force field (such as AMBER^14^, CHARMM^15^, GROMOS^16^, or OPLS-AA^17^) is an alternative to study the interaction mechanism between the aptamer and the target molecule on the atomic scale. As an example, Rhinehardt used molecular dynamics (MD) simulation to explore the stable binding sites of three DNA aptamers on interleukin (IL6)^18^. The radius of gyration (Rg), the center of mass (COM) distance, and the number of intermolecular hydrogen bonds was used to determine whether the complex of aptamer and IL6 is stable. In Cunzheng’s study^19^, they used the molecular dynamics and molecular docking simulation to investigate the interaction between the DNA aptamer-combined molecular beacon probes (MB) and organophosphorus pesticides. Their simulation results show that the two main factors promoting the stable interaction of aptamer/organophosphorus pesticides are the intermolecular hydrogen bonding and the Van der Waals energy. In Yan's study^20^, the quality of the aptamer was examined both by considering the thermodynamic stability and by determining the kinetic residence time when the aptamer binds to its target molecule. The docking simulation results from AutoDock show that the residence time is significantly related to the aptamer’s affinity towards the target molecule.

To expand the temporal and spatial domains in biomolecular simulations, different coarse-grained (CG) models and force fields have been developed. For example, in Markegard’s study^21^, they developed a CG model, named BioModi, to observe the effect of temperature on the large-scale DNA self-assembly process. Their simulation results show that a large number of DNA clusters form at a moderate temperature, while the cluster size decreases at high temperature. This phenomenon is also consistent with the experimental observation. In Maffeo’s study^22^, they proposed a CG model for DNA, by which the backbone and sugar/base groups of nucleotides are coarsely grained to two interaction sites. The strength of this model is in its reproduction of an experimentally-measured force-extension profile for an unstructured DNA strand. In the CG model proposed by Poulain^23^, their strategy coarsely grains the phosphate backbone into one bead, the sugar ring into two beads, and the nucleobases into two or three beads (two for cytosine/thymine; three for adenine/guanine). The protein–DNA docking process was simulated by this CG method and the results show this CG model can predict the protein–DNA complex structure as that predicted by the all-atom model. The results by both CG and AA models are also very close to the one in the protein data bank. They also indicate the CG van der Waals parameters between A, G, C and T possess the sensitivity to different DNA sequences, which are sufficient for detecting DNA sequence preferences.

In Monticelli’s study on the CG model^24^, the MARTINI force field was first proposed to simulate the lipid and membrane systems, with results exhibiting very similar structural and dynamical behaviors as those predicted by the all-atom models. Accordingly, the MARTINI force field has been widely used to predict the stable structures in specific solutions for vesicle^25–27^, micelle^28^, lipid bilayer^29^, and nanodisc systems^30^. In Monticelli’s further study^31^, the MARTINI force field was extended to model the interaction of bio-molecules including protein, DNA, and RNA. Compared with the structures predicted by the all atom model, it has been confirmed that the MARTINI force field not only accelerates the simulation process by about three orders, but also preserves the characteristics of different amino acids, as the all atom model also does. The comparisons between the structures predicted by the MARTINI CG and all atom models can be also seen for a peptide-bilayer system^32^, DNA^33^, and DNA–protein complexes^34^, which show good reproductively of the CG model to those by the AA model.

In our previous studies^35,36^, we have developed and improved a global minimum search method for the all atom model in vacuum, the stochastic tunnelling-basin hopping-discrete molecular dynamics (STUN-BH-DMD) method. For modelling a system in the water environment, the STUN-BH-DMD method was extended to a coarse-grained system in the current study. It can identify the low-lying binding configuration of a larger molecule on a target molecule with a high calculation efficiency. The biomolecule complex of an epithelial cell adhesion molecule (EpCAM) with the single-stranded DNA aptamer EpA (Apt_EpA_, 5′-ACA GAG GTT GCG TCT GT-3′) in vacuum was obtained using the AA mode with the AMBER99sb force field^37^. If the STUN-BH-DMD method were directly applied to a system matching the experimental environment by the AA model, the large number of water molecules in the simulation makes the computational capacity required to be several orders higher than that in a vacuum, which makes STUN-BH-DMD impractical. Since the MARTINI CG model can accelerate the simulation process by about three orders faster than that of the corresponding AA model, this model was used with STUN-BH-DMD to find the most stable Apt_EpA_/EpCAM complex in a water environment. Then the Apt_EpA_/EpCAM complex was relaxed in the water by a long-term CGMD simulation. During the CGMD simulation, the root mean square deviation (RMSD) and the Rg were used to monitor the stability of EpCAM. For Apt_EpA_, the root mean square fluctuation (RMSF) analysis was also used to indicate the flexibility of each Apt_EpA_ nucleobase during the last 100 ns of the CGMD simulation**.**

## Simulation model

**Figure 1:**
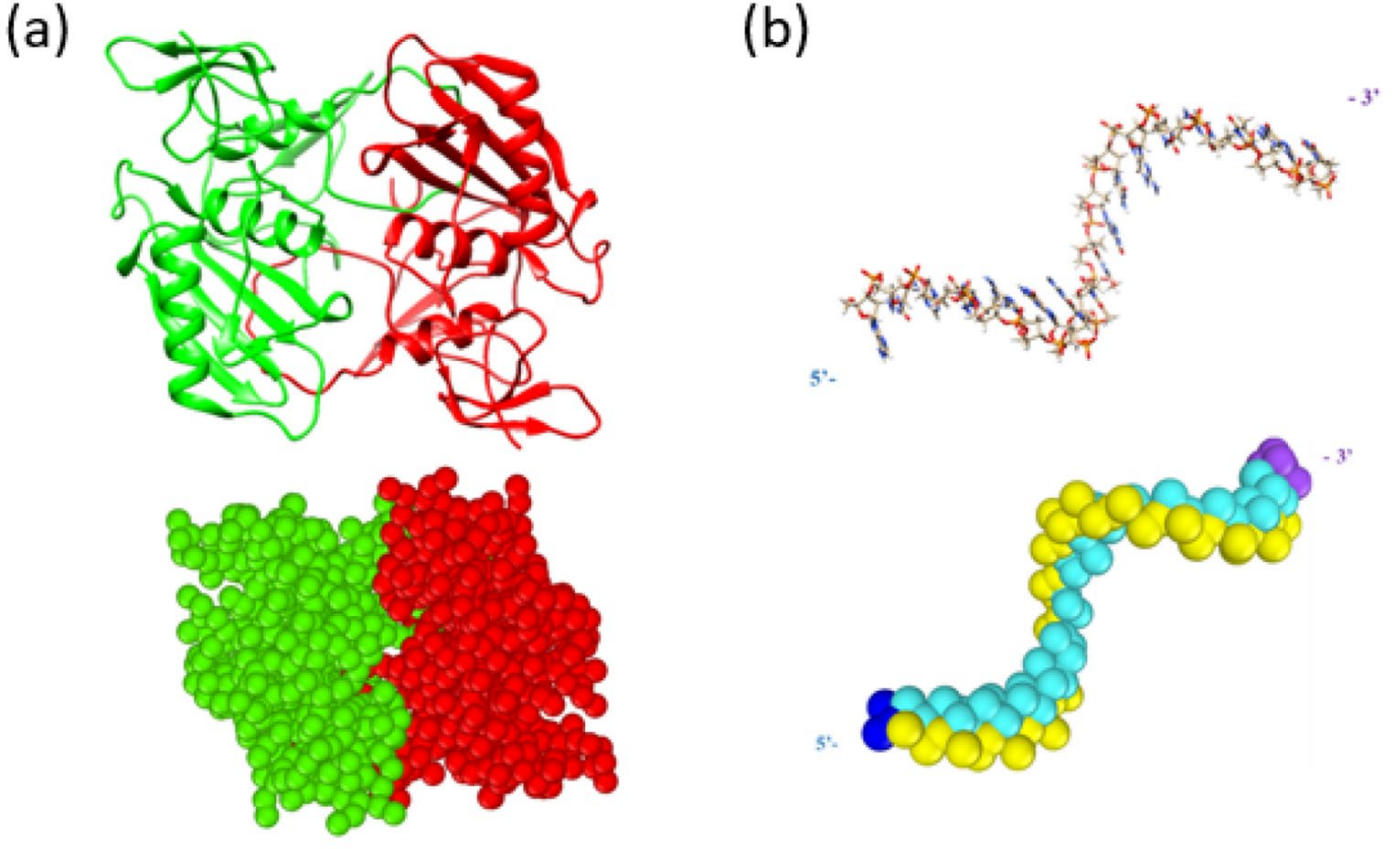
The upper panels show the all atom model and the lower panel show the CG model using the MARTINI force field for (**a**) the structure of epithelial cell adhesion molecule (EpCAM) dimer and (**b**) the EpA aptamer (Apt_EpA_) with the sequence 5′-ACA GAG GTT GCG TCT GT-3′. The Apt_EpA_ backbone and nucleobase are shown in yellow and cyan, while the 5′ and 3′ beads are colored in dark blue and purple.

To construct the EpCAM and Apt_EpA_ CG models, a python script developed by de Jong and Wassenaar^38^ were used to convert the AA models to their corresponding CG models according to the MARTINI coarse-graining strategy. The AA structure of EpCAM was constructed by using the pdb file (PDB ID: 4MZV^39^) in the Protein Data Bank (PDB)^40^. The upper panel of Fig. 1a shows an illustration of EpCAM based on the AA model, where it can be seen that EpCAM contains two structural domains of a dimer having a total number of 7610 atoms, including secondary structures of alpha helixes, beta sheets, and loops. The EpCAM dimer is constructed by winding looped fragments of each EpCAM molecule. After converting the AA model to CG model, the corresponding EpCAM CG structure with 1068 CG beads is shown in the lower panel of Fig. 1a.

In Macdonald's study^41^, it was reported that the aptamer EpA (designated as Apt_EpA_), with the 5′-ACA GAG GTT GCG TCT GT-3′ sequence, possesses the ability to recognize EpCAM in the water environment. Therefore, Apt_EpA_ was used in this study to explore the most stable binding configuration on EpCAM in the water environment. The upper and lower panels of Fig. 1b depict the Apt_EpA_ in the AA model with 543 atoms and its corresponding CG model with 110 CG beads, respectively.

The stochastic tunnelling-basin hopping-discrete molecular dynamics (STUN-BH-DMD) global minimum search method was used to find the most stable binding configuration of Apt_EpA_ on the EpCAM in the water environment for current MARTINI CG model. The detailed introduction of STUN-BH-DMD method for the all-atom method can be found in our previous studies^35,36^, and the following several paragraphs are the introduction about how the STUN-BH-DMD method was applied to the current CG system.

Calculating the energy and changing the atomic coordinates are two basic steps for the STUN-BH-DMD search iteration and the system evolution was conducted by evaluating the Boltzmann factor. In the STUN method, the MD simulation at a higher temperature and with a larger integration time step was used to change the atomic coordinates. However, the closest local minimum structure after the MD step cannot be obtained if the original STUN method is used. Consequently, the molecular statics using conjugate gradient optimization in the BH method was used to obtain the closest local minimum structure after the MD part of STUN^35,36^. In the original BH method for finding the most stable LJ nanocluster, the atom coordinates were randomly moved for the next search step. However, for the current Apt_EpA_/EpCAM system, the random movements of atomic coordinates could cause overlap between Apt_EpA_ and EpCAM, which produce a higher probability of rejection after the evaluation of the Boltzmann factor. Accordingly, the MD part of the STUN method was used to replace the random atom movement in the original BH method^35,36^.

During the MD process, Apt_EpA_ fragments that interact more strongly with the EpCAM could undergo a very small orientation change compared to fragments with higher interaction energy. To extend the local spatial domain search, the DMD was also used with MD to change the atomic coordinates during the search process. DMD uses a step potential function to maintain repulsive interactions when the distance between two atoms is shorter than a threshold value, as well as to significantly reduce the interaction strength between two atoms when the distance between them exceeds a threshold value^35,36^. Although the advantage of DMD is to expand the spatial search domain, it could also destroy the local stable configuration built into the previous MD steps, and moreover, it cannot find a more stable configuration. Consequently, MD and DMD were iteratively used during the STUN-BH-DMD search process through which MD was conducted in a STUN-BH-DMD iteration, and DMD was performed in a subsequent STUN-BH-DMD iteration^35,36^. By combining the advantages of MD and DMD, the STUN-BH-DMD method possesses the ability for a wider spatial domain search for the Apt_EpA_ orientation to the EpCAM as well as retaining the local Apt_EpA_ fragment which has relatively stronger energy with the EpCAM^35,36^.

The effective potential ${\text{f}}_{\text{STUN}}(\text{x})$ has the following formula:

1 $${E}_{STUN}(x)=\text{ln}\left(\left(f\left(x\right)-{f}_{0}\right)+\sqrt{{\left(f\left(x\right)-{f}_{0}\right)}^{2}+1}\right)$$

where $x$ stands for the coordinates of atoms, and ${f}_{0}$ is the lowest binding energy formed by the Apt_EpA_ bases and EpCAM amino acids obtained thus far. The value of ${f}_{0}$ in Eq. (1) is replaced when a lower binding energy (i.e., when the ${E}_{STUN}(x)$ value is negative) than the current ${f}_{0}$ value is found during the following STUN-BH-DMD search^35,36^. Furthermore, *f(x)* is the interaction energy between the Apt_EpA_ bases and EpCAM amino acids at the atom coordinates *x*. This effective potential preserved all minima locations lower than ${f}_{0}$, and the entire energy space from ${f}_{0}$ to the potential maximum was mapped onto the interval greater than 0^42^. The binding energy ${G}_{BE}$ was calculated according to Eq. (2):

2 $${G}_{BE}={G}_{EpA/EpCAM}-{G}_{EpA}-{G}_{EpCAM}$$

where ${G}_{EpA/EpCAM}$, ${G}_{EpA}$, and ${G}_{EpCAM}$ are the potential energies of aqueous systems with both EpA and EpCAM, with isolated EpA only, and with isolated EpCAM only, respectively^35,36^. The effective potential energy of STUN shown in Eq. (1) converts the original potential energy surface (PES) into a smoother potential energy surface that allows the configuration to tunnel the forbidden regions for a wider spatial search. The effective potential energy surface still keeps the same local minimum structure as those within the original potential energy surface. Consequently, the BH part of STUN-BH-DMD method uses the conjugate gradient method to conduct the geometrical optimization of the Apt_EpA_/EpCAM complex^35,36^. Then the binding energy of the optimized structure by MARTINI force field was used to obtain the effective energy by Eq. (1) for the Boltzmann factor evaluation.

The difference between DMD and traditional MD lies in the potential energy function of their interactions. In the original DMD, the step potential function was used for the hard sphere interaction, which makes atoms repulsive when they are close to each other; otherwise, no interaction was considered. Consequently, atoms could have their relative coordinates changed more easily by DMD than by traditional MD. In the STUN-BH-DMD method, we did not use a step function for the DMD part, and the interaction strengths of nonbonding interactions (including the Lennard–Jones and Coulombic interactions) were reduced to 1/100 times smaller than their original MARTINI parameters^35,36^. Thus, the DMD interaction between beads works in a highly similar manner to those using a step potential function.

A total of 4000 independent initial Apt_EpA_ orientations to EpCAM were considered for enhancing the global spatial domain search. In order to generate 4000 different Apt_EpA_ orientations to EpCAM, EpCAM was randomly rotated at its center of mass for 4000 times, while the Apt_EpA_ was kept at the same coordinates for each EpCAM random rotation. One hundred STUN-BH-DMD iterations were performed for each initial orientation configuration. A total of 400,000 STUN-BH-DMD iterations were performed to facilitate the search for the most stable adsorption configuration between Apt_EpA_ and EpCAM. For each STUN-BH-DMD iteration, the fire optimization was conducted by following the NVT MD or DMD simulation at 600 K for 300 steps (3 ps) to extend the local spatial search domain^35,36^. The energy of optimized structure using the CG method was used as *f(x)* for Eq. (1), and thus the subsequent interaction between Apt_EpA_ and EpCAM could be performed through STUN to find a lower energy configuration. If the value of ${f}_{STUN}(x)$ is negative, which means that *f(x)* is smaller than ${f}_{0}$ and the energy value of *f(x)* is stored as ${f}_{0}$. The structure is preserved and the STUN-BH-DMD process evolves in the direction of the lower energy. If the current ${E}_{STUN}(x)$ is greater than the value of ${\text{STUN}}_{\text{last}}$ (the last ${E}_{STUN}\left(x\right)$ stored after Boltzmann factor evaluation) recorded by the system, it will be determined by generating a random number from 0 to 1 as the acceptance ratio and calculating the Boltzmann factor^35,36^. If the value of the Boltzmann factor is greater than the receiving ratio, it is accepted; otherwise, the current structure is skipped, and the preferred configuration searched for in the previous one is advanced. The Boltzmann factor has the following formula^35,36^:

3 $$\text{Boltzfactor}=\text{exp}\left[\frac{{\text{STUN}}_{\text{last}}-{E}_{STUN}(x)}{kT}\right]$$

1. If ${E}_{STUN}(x)$ is lower than or equal to ${\text{STUN}}_{\text{last}}$, this STUN-BH-DMD step is accepted. The ${\text{STUN}}_{\text{last}}$ is renewed by the current ${E}_{STUN}(x)$ and xlast (the atom coordinates at ${\text{STUN}}_{\text{last}})$ is also replaced by the current atom coordinates.
2. If ${E}_{STUN}(x)$ is greater than ${\text{STUN}}_{\text{last}}$, a random number between 0 and 1 is generated and the Boltzmann factor is determined according to Eq. (3). If the Boltzmann factor is greater than the random number, this STUN-BH-DMD step is accepted. The ${\text{STUN}}_{\text{last}}$ is renewed by the current ${E}_{STUN}(x)$ and xlast is also replaced by the current atom coordinates.
3. If ${E}_{STUN}(x)$ is greater than ${\text{STUN}}_{\text{last}}$ and the corresponding Boltzmann factor is smaller than the random number generated between 0 and 1, this STUN-BH-DMD step is rejected. The system atom coordinates are replaced by xlast for the next STUN–BH step.

The value of kT shown in Eq. (3) was dynamically adjusted every 20 STUN-BH-DMD steps to maintain the acceptance percentage close to 50%^35,36^. The STUN-BH-DMD method can significantly guide the system to evolve in the direction of lower energy.

**Figure 2:**
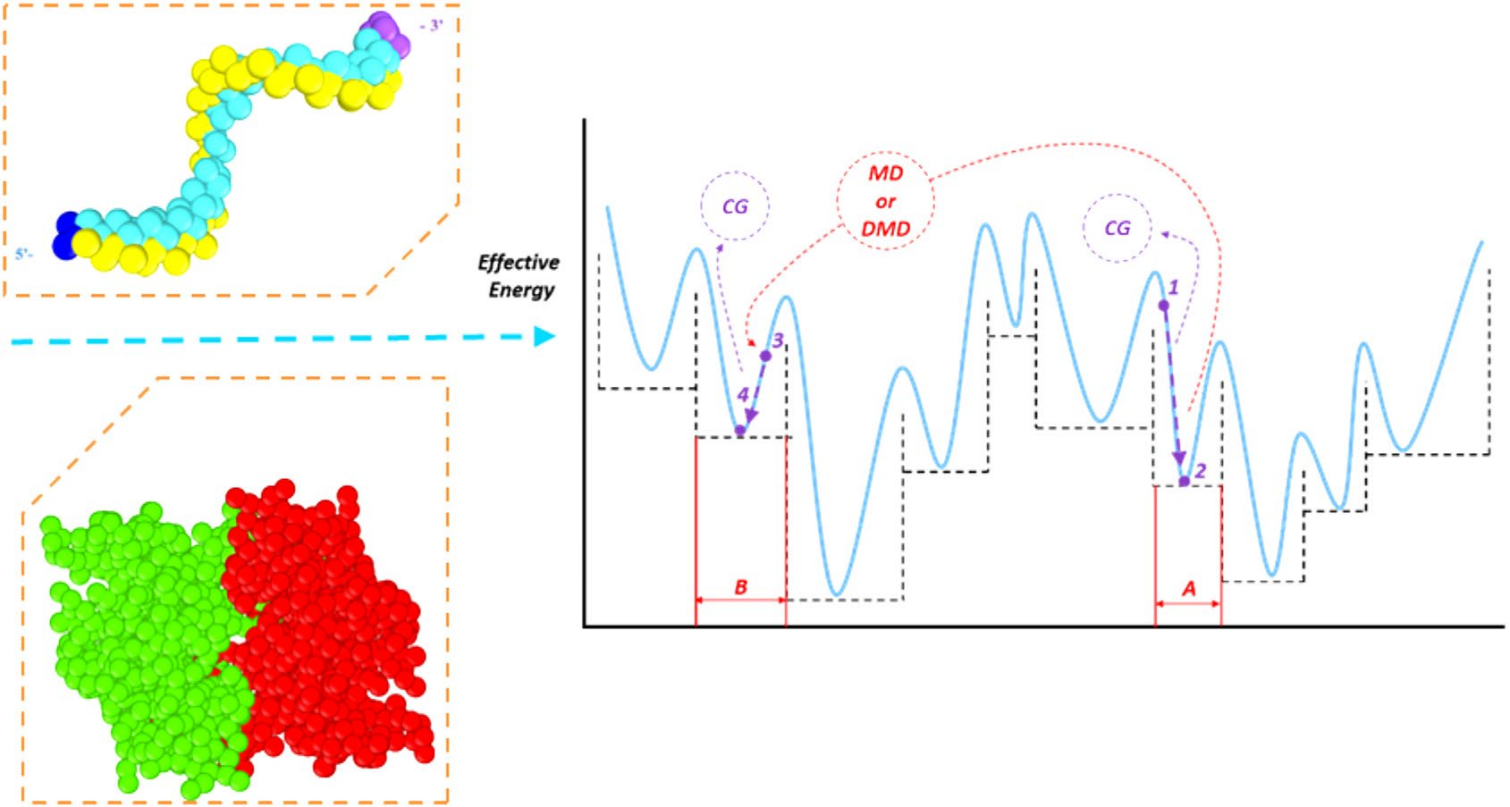
Schematic of the STUN-BH-DMD method for MARTINI CG model. (**A**, **B**) represent two energy basins (Basin A and Basin B) of the effective potential energy surface; Points 1 and 3 stands for the structures with the lowest effective potential energies at Basin A and Basin B obtained by the MARTINI CG method, respectively. Points 2 and 4 are the structures located at Basin A and Basin B, respectively.

Figure 2 summarizes the global minimum search process using the STUN-BH-DMD method, which is also used for the AA model in our previous studies^35,36^. The binding energy formed between the Apt_EpA_ bases and EpCAM was first converted to the effective energy according to the function in the STUN method. The initial configuration was assumed to correspond to the effect energy at Point 1 within Basin A, and then molecular statics using the conjugate gradient (CG) method (as adopted in the BH method) was used to quickly find the nearest local minimum structure at Point 2 within Basin A, leading to the system evolving from Point 1 to Point 2, seen in Fig. 2. Using the CG method, all structures within Basin A corresponded to the same local minimum structure at Point 2 of Basin A. This indicated that using the CG method can convert the effective energy of each basin into the flat PES^35,36^, as shown by the dashed horizontal line.

Next, MD or DMD were performed for 300 steps for the Apt_EpA_ configuration at Point 2 to generate more Apt_EpA_ orientations for the target EpCAM to jump to another basin, as shown in Fig. 2, where the configuration at Point 2 in Basin A changed to that at Point 3 in Basin B. Subsequently, the local minimum structure at Point 4 in Basin B could be quickly found using the CG optimization. Many local minimum structures in different basins can be quickly obtained by repeating the abovementioned steps and compared with the previously calculated values of the stable structure using the Boltzmann factor^35,36^. The configuration search is guided towards the direction of lower effective energy, and finally the most stable structure (global minimum structure) can be found.

For constructing the STUN-BH-DMD simulation system, Apt_EpA_ and EpCAM were first placed into the simulation box, within which the closest distance between Apt_EpA_ and EpCAM is about 20 Å. Then the water molecules were randomly inserted into the system until the system density was close to 1 g/cm^3^. A total of 19,852 water beads were randomly inserted into the simulation box with a closest distance of 5 Å allowed between any beads of AptEpA and EpCAM. Twenty cations were also randomly inserted into the system to neutralize the simulation system. After the STUN-BH-DMD search, the selected Apt_EpA_/EpCAM complexes underwent the canonical ensemble (NVT) by the Berendsen thermostat at 300 K for 500 ns. The Lennard–Jones potential was smoothly shifted to zero between a distance 9 Å and the cutoff distance of 12 Å. The Coulombic potential was also smoothly shifted to zero between a distance 0.5 Å and the cutoff distance of 12 Å. Details of simulation parameters can be seen in Table 1.

**Table 1:** The STUN-BH-DMD simulation parameters for the MARTINI CG model.

Simulation parameters	Value or number
Density	1.04 g/cm^2^
Simulation box (l_x_* l_y_* l_z_)	119.2 Å*129.2 Å*172.7 Å
Dielectric constant	15
Time step size (MD and DMD)	10.0 fs
Temperature (MD and DMD)	600 K
Apt_EPA_	110 beads
EpCAM	1068 beads
Standard water (P4)	19,852 beads
Antifreeze (BP4)	2206 beads
Cation (Qd)	20 beads

All molecular simulations including STUN-BH-DMD method and MD simulations were conducted using the Large-scale Atomic/Molecular Massively Parallel Simulator (LAMMPS) package^43^, utlizing the MARTINI force field (version 2.2)^38^, in order to describe the bead interactions for the Apt_EpA_/EpCAM CG system. Chimera (version 1.13.1)^44^ and OVITO (version 3.3.5)^45^ were used to depict these molecular structures as well as to perform the post-processing.

## Result and discussion

**Figure 3:**
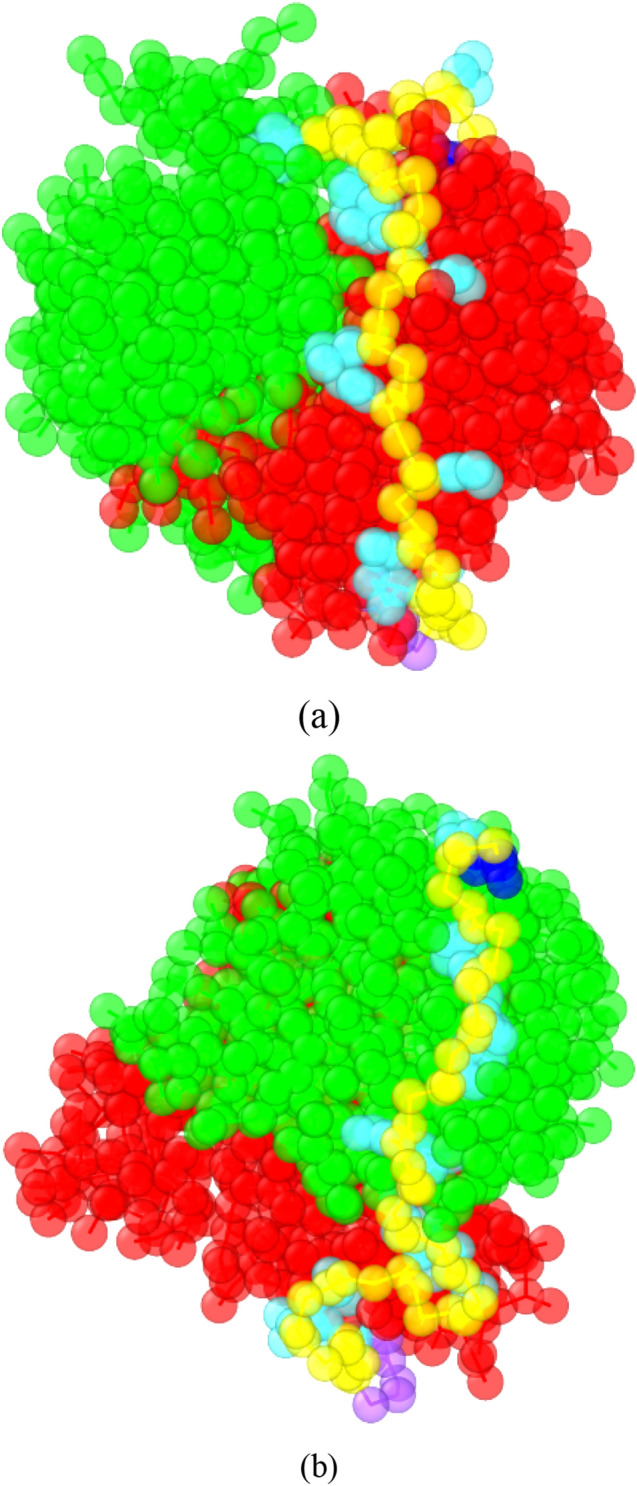
The CG Apt_EpA_/EpCAM complex structures with the two lowest binding energies after the STUN-BH-DMD search for (**a**) CG_Case 1 and (**b**) CG_Case2. The Apt_EpA_/EpCAM binding energies of CG_Case1 and CG_Case2 are about − 1837.8 kJ/mol and − 1675.1 kJ/mol, respectively. The Apt_EpA_ backbone and nucleobase are shown in yellow and cyan, while the 5′ and 3′ beads are colored in dark blue and purple. The molecules of the EpCAM dimer are coloured in green and red, respectively. For clarity, all water beads, antifreeze beads, and ionic Na+ beads are hidden.

Figure 3a,b show the CG Apt_EpA_/EpCAM complex structures with the two lowest binding energies. The STUN-BH-DMD method was directly implemented on Apt_EpA_ and EpCAM in the water environment. For convenience in presenting our simulation results, these two CG Apt_EpA_/EpCAM complex structures are designated as CG_Case1 and CG_Case2, which possess the corresponding binding energies about − 1837.8 kJ/mol and − 1675.1 kJ/mol, respectively. One can see from Fig. 3 that there are two most stable Apt_EpA_ binding configurations on EpCAM, and the binding energy of CG_Case2 is about 8.9% higher than that of CG_Case1. The Apt_EpA_ binding conformation on EpCAM of CG_Case1 is very similar to the most stable adsorption configuration by the AA model in vacuum^35^.

**Figure 4:**
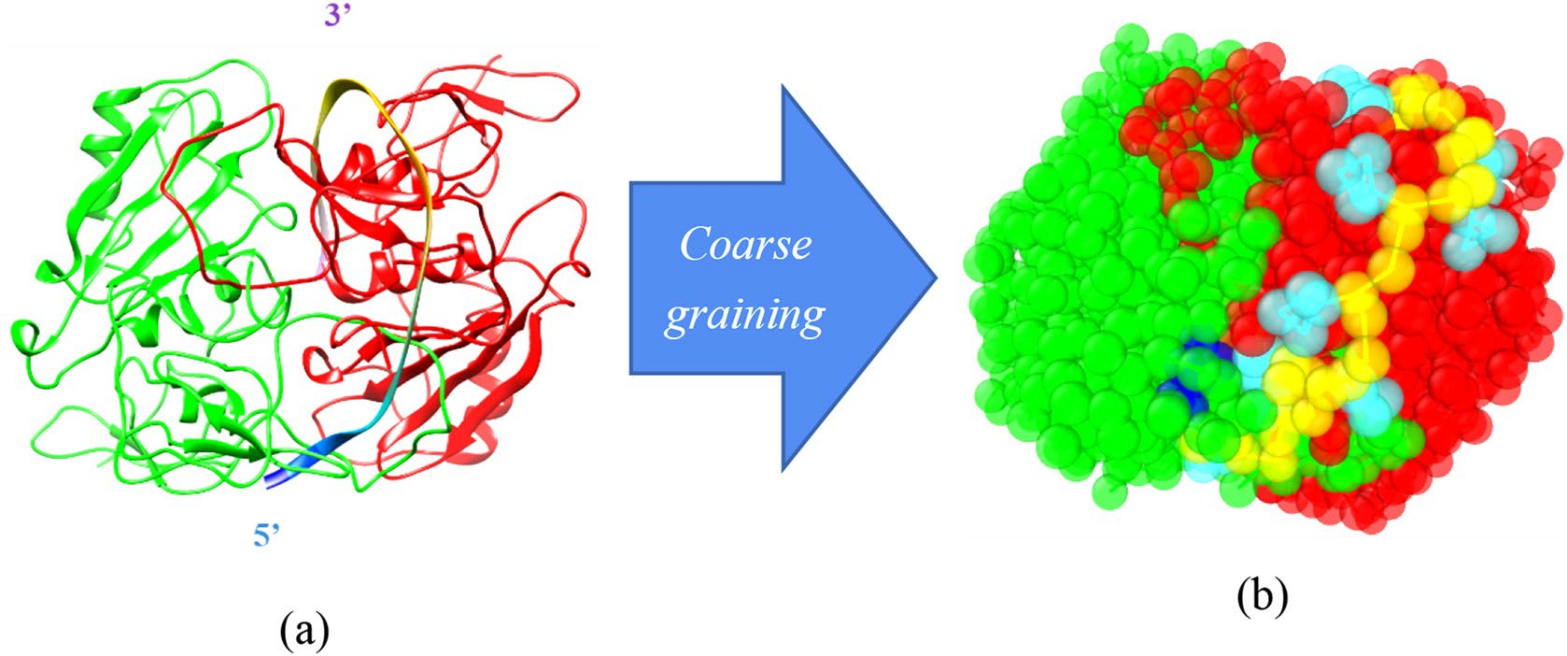
(**a**) The all atom model and (**b**) the corresponding MARTINI CG model for the most stable Apt_EpA_/EpCAM complex in vacuum. The Apt_EpA_ backbone and nucleobase are shown in yellow and cyan, while the 5′ and 3′ beads are colored in dark blue and purple. The EpCAM dimer are colored in green and red, respectively.

To better understanding the difference in the Apt_EpA_ binding mechanism in vacuum and in water, the most stable AA Apt_EpA_/EpCAM complex structure obtained in our previous study^35^ was coarse-grained according to the MARTINI CG model. It should be noted the Amber99SB force field was used for this AA result and using a newly-developed force field could obtain a more reliable adsoprtion configuration. This CG Apt_EpA_/EpCAM is directly converted from the Apt_EpA_/EpCAM AA model with the lowest binding energy, and is designated as CG_AA. Figure 4 shows the AA model and the corresponding CG model for CG_AA. After the relaxation by the molecular statics in vacuum, this relaxed CG_AA was relaxed again in the CG water environment using the simulation parameters listed in Table 1. After the minimization by the conjugate gradient algorithm, the calculated Apt_EpA_/EpCAM binding energy is about − 1780 kJ/mol. The binding energy of CG_AA equilibrated in water is still higher than that of CG_Case1 by 3.1%. It also indicates the STUN-BH-DMD process directly implemented for the water environment can determine the most stable Apt_EpA_/EpCAM complex structure in water, which matches the experimental environment more closely.

**Figure 5:**
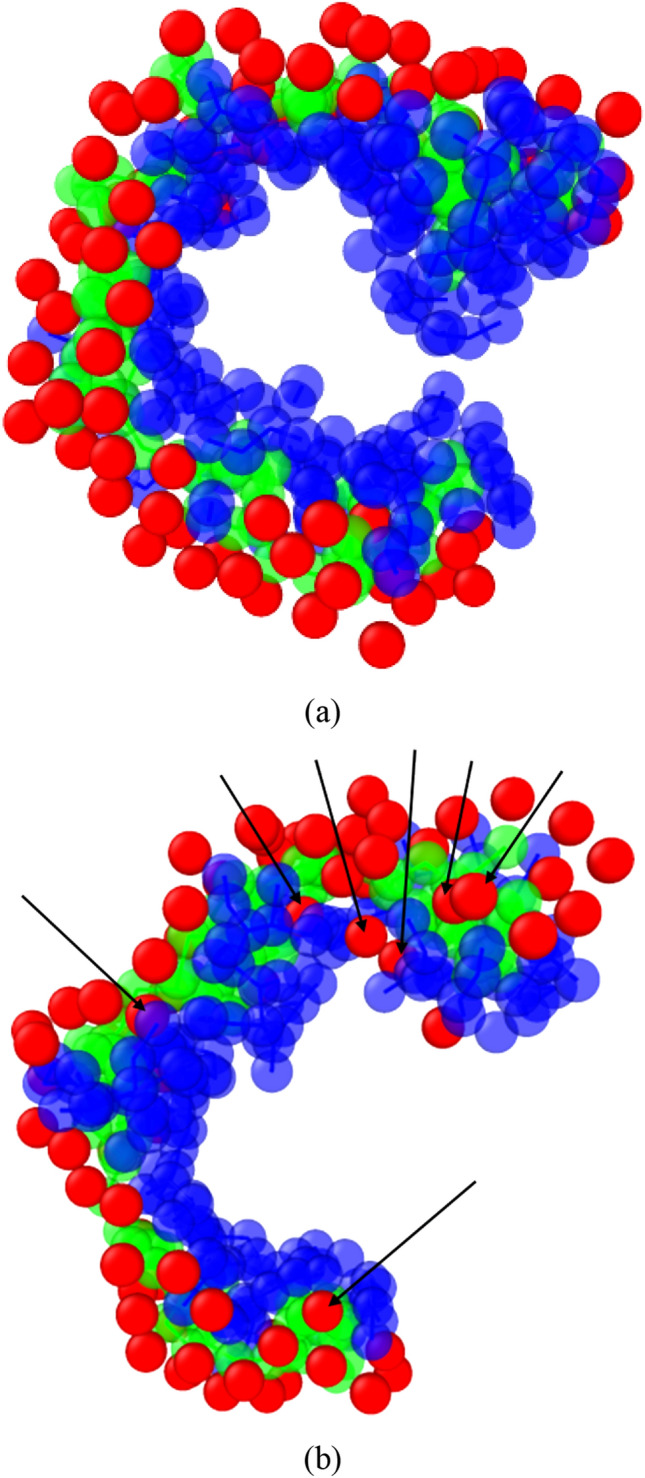
The water bead distribution within 7 Å from Apt_EpA_ for (**a**) CG_AA and (**b**) CG_Case1. The arrows indicate the water beads located at the interface between Apt_EpA_ and EpCAM. The Apt_EpA_, EpCAM, and water beads are marked in green, dark blue, and red, respectively. For clarity, the rest water beads, antifreeze beads, and Na^+^ beads are hidden.

To further investigate the role of water on the Apt_EpA_/EpCAM complex structure for Case_AA and CG_Case1, Fig. 5 shows the water beads and EpCAM beads distributed within 7 Å from Apt_EpA_. For clarity, the Apt_EpA_, EpCAM, and water beads are colored in green, dark blue, and red, respectively. For CG_AA as shown Fig. 5a, one can see there are no water beads located at the interface between Apt_EpA_ and EpCAM. Because the Apt_EpA_/EpCAM complex structure of CG_AA was first predicted by the STUN-BH-DMD method in vacuum, the Apt_EpA_ has occupied the stable adsorption sites of EpCAM. Even though the Apt_EpA_/EpCAM complex structure of CG_AA was further relaxed in the water environment, the water beads still cannot occupy the EpCAM sites, as they are blocked by Apt_EpA_ beads. For CG_Case1 shown in Fig. 5b, the arrows indicate the water beads are located at the interface between the Apt_EpA_ and EpCAM beads. Before conducting the STUN-BH-DMD method for searching stable Apt_EpA_/EpCAM complex structures, both Apt_EpA_ and EpCAM were hydrated and relaxed by interacting with their surrounding water beads. Consequently, water beads occupy all surface sites of Apt_EpA_ and EpCAM before Apt_EpA_ approaches EpCAM. As Apt_EpA_ approaches EpCAM and then interacts with it, some water beads on the EpCAM are excluded by Apt_EpA_. Because terminal fragments of Apt_EpA_ possess higher flexibility than does the middle fragment, water beads tend to reside in the area between the Apt_EpA_ terminal and EpCAM. These water beads near the Apt_EpA_ terminal also stabilize the interaction between these Apt_EpA_ terminals and EpCAM, indicating that water molecules play an important role when binding occurs in an aqueous solution.

**Figure 6:**
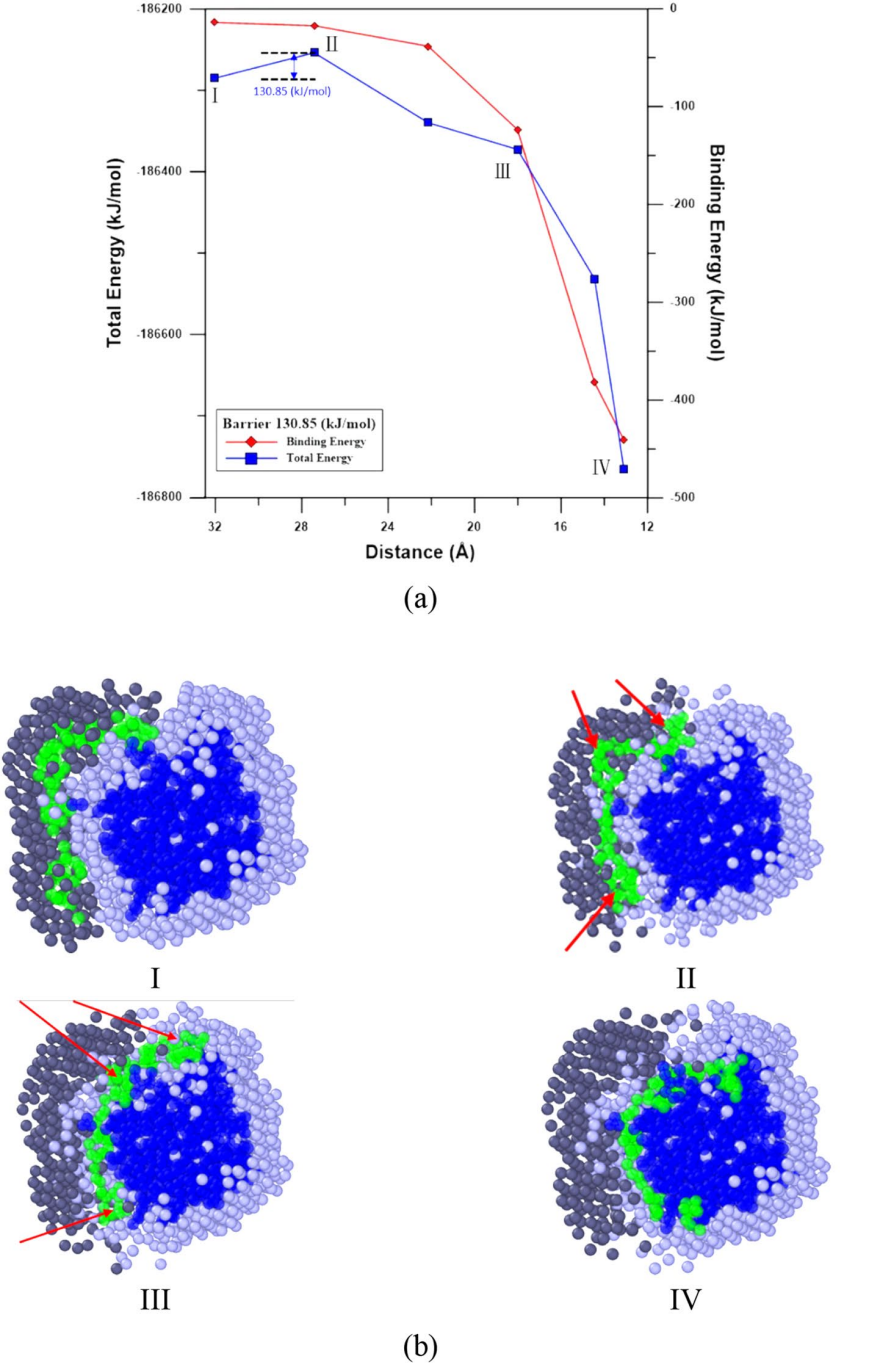
(**a**) Total energy and binding energy profiles with the center-of-mass distance between Apt_EpA_ and EpCAM for CG_Case1 during the NEB process. (**b**) The slice plane views of water bead distribution within 10 Å from Apt_EpA._ Morphologies I to IV correspond to structures as labelled in the NEB profile. The Apt_EpA_, EpCAM, water beads surrounding Apt_EpA_ and water beads surrounding EpCAM are marked in green, dark blue, dark gray, and light gray, respectively.

**Figure 7:**
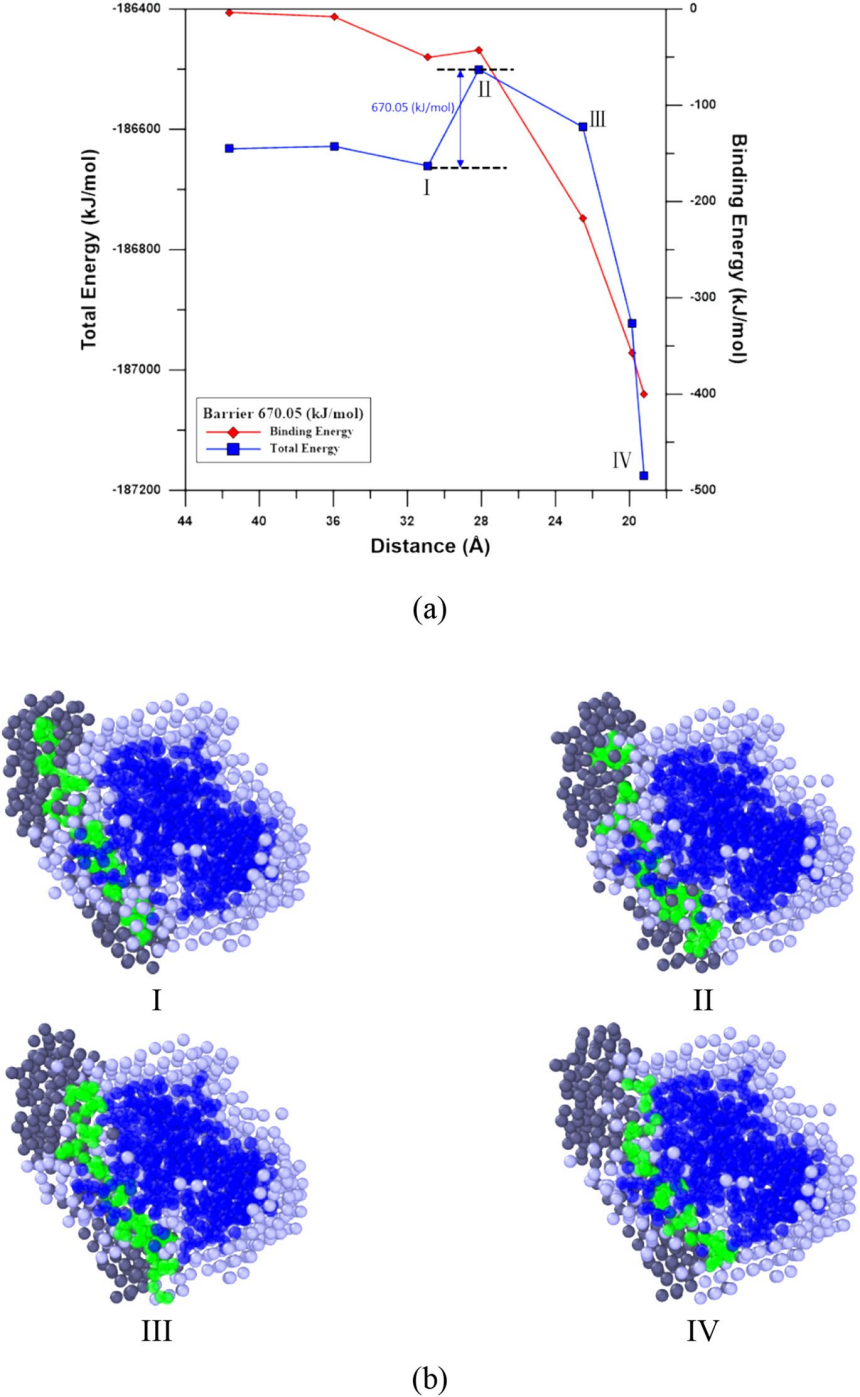
(**a**) Total energy and binding energy profiles with the center-of-mass distance between Apt_EpA_ and EpCAM for CG_Case2 during the NEB process. (**b**) The slice plane views of water bead distribution within 10 Å from Apt_EpA._ Morphologies I to IV correspond to structures as labelled in the NEB profile. The Apt_EpA_, EpCAM, water beads surrounding Apt_EpA_ and water beads surrounding EpCAM are marked in green, dark blue, dark gray, and light gray, respectively.

To explore the Apt_EpA_ adsorption process near its stable adsorption sites on EpCAM, the nudged elastic band (NEB) method^46,47^ with 16 images implemented by LAMMPS was used to find the minimum energy pathway (MEP) of Apt_EpA_ adsorption for CG_Case1 and CG_Case2. In Bell’s study^48^, the EpCAM aptamer, EP23 (an RNA with sequence ACGUAUCCCUUUUCGCGUA) was used to understand its binding affinity to the EpCAM dimer. The EP23 was first folded according to the Mfold prediction^49^, and then the four most stable EP23/EpCAM complexes found by the docking simulation were considered. The steered molecular dynamics (SMD) simulation was used to simulate the pulling process of EP23 from EpCAM, which includes the conformational change of EP23 along the assumed reaction pathway corresponding to the inverse process of the EP23 adsorption process to EpCAM. Consequently, for the Apt_EpA_/EpCAM complex, the pulling processes of Apt_EpA_ from EpCAM for CG_Case1 and CG_Case2 by SMD were conducted to prepare reasonable NEB reactant image structures. The relaxed Apt_EpA_/EpCAM complexes in the water environment shown in Fig. 3a,b were used for the product image structures of NEB. Figures 6 and 7 show the MEPs of Apt_EpA_ adsorption process onto EpCAM, as well as the corresponding Morphologies I to IV as labeled on the MEP profiles. In Figs. 6a and 7a, both the total energies of the system and the binding energies between Apt_EpA_ and EpCAM were shown with decreasing distance between the mass centers of Apt_EpA_ and EpCAM. The MEP profiles shown in Figs. 6a and 7a start from the NEB image, where the interaction between Apt_EpA_ and EpCAM begins and the binding energies become lower than 0. To investigate the Apt_EpA_ conformational change and the water redistribution during the adsorption process, in Figs. 6b and 7b, the Apt_EpA_ and EpCAM beads are marked in green and dark blue, while the water beads around Apt_EpA_ and around EpCAM are marked in dark gray and light gray, respectively. All water beads at Morphology I were used as referenced water beads for Morphologies II–IV. For CG_Case1, the MEP profile shown in Fig. 6a indicates the Morphology II is a transition state from Morphology I with a barrier of about 130.8 kJ/mol. Once the mass center distance is shorter than that at Morphology II, both total energy and the binding energy display a distinct drop. From Morphologies I–IV shown in Fig. 6b, one can see the water beads around EpCAM were gradually excluded by Apt_EpA_ when Apt_EpA_ approaches EpCAM. The excluded water beads were then redistributed with the water beads around Apt_EpA_. At Morphology II, the arrows indicate the significant conformational change of Apt_EpA_ takes place, compared with that at Morphology I. Although the Apt_EpA_ begins to interact with EpCAM at Morphology II, this binding energy is not sufficient to overcome the energy barrier for the Apt_EpA_ conformational change. For CG_Case2 shown in Fig. 7a, the transition structure at Morphology II has an energy barrier of about 670.05 kJ/mol to overcome (from Morphology I) in order to reach the stable Apt_EpA_/EpCAM complex at Morphology IV. From Fig. 7b, the Apt_EpA_ conformation does not undergo a significant change during the adsorption process, so it infers the energy barrier mainly comes from the exclusion of water beads residing on EpCAM when the Apt_EpA_ continuously approaches EpCAM.

**Figure 8:**
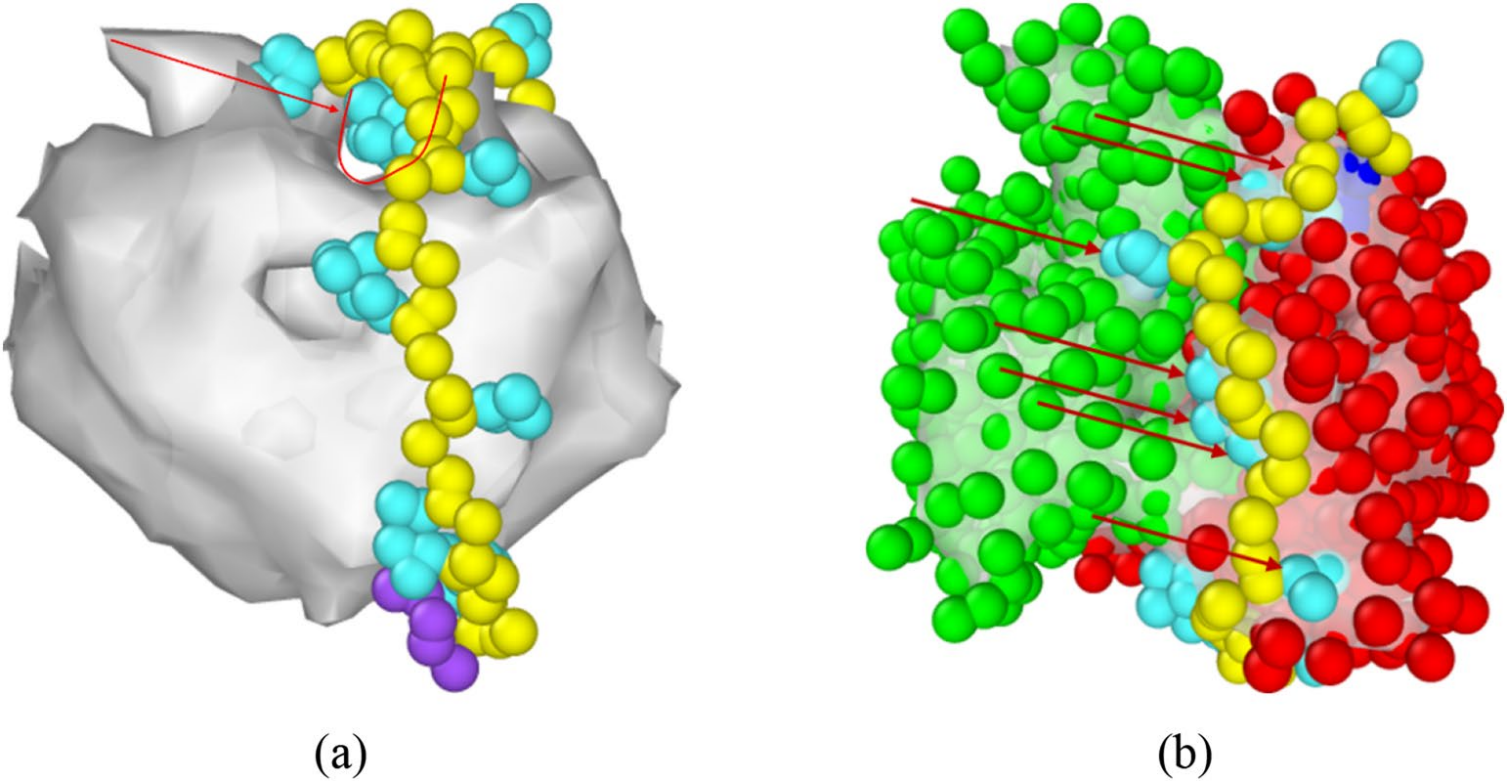
(**a**) The surface mesh of EpCAM with Apt_EpA_ beads for CG_case1. The arrow and the U-shape symbol demonstrate the pocket space formed by the EpCAM dimer; (**b**) the EpCAM surface mesh, EpCAM beads, and Apt_EpA_ beads for CG_case1. The arrows indicate the Apt_EpA_ fragment (from base 3 to base 9) within the EpCAM pocket. The Apt_EpA_ backbone and nucleobase are shown in yellow and cyan, while the 5′ and 3′ beads are colored in dark blue and purple. The two sides of the EpCAM dimer are shown with green and red. For clarity, all water beads, antifreeze, and ionic Na^+^ are hidden.

In Hayashi’s study^50^, they used MD simulation to explore the important role of water molecules on the molecular recognition for a folded RNA aptamer R12 (with the sequence GGAGGAGGAGGA) on P16 (partial peptide of a prion protein). In aqueous solution, R12 and P16 are hydrated before binding. Water molecules around these two molecules form the exclusion volumes (EVs), within which water molecules interact more strongly with R12 and P16 than they do with those outside the exclusion volume. During the binding process, the overlap of R12 and P16 exclusion volumes become more significant and cause dehydration in the overlap regime, leading to an increase in the interaction energy between the water and these molecules. Accordingly, the system energy also increases during the dehydration process. At the same time, water molecules leaving the EV are reoriented with those outside the EVs to form more stable water-water arrangement, resulting in the decrease in system energy. If the binding molecules are more flexible, the significant structural change leads to a larger decrease in the total EV and a large gain of the configurational entropy of water (entropic EV effect) after the complex forms. Figure 8a shows the surface mesh of the EpCAM dimer and Apt_EpA_ beads by OVITO. The arrow and the U-shape symbol demonstrate that there is a pocket space formed by the EpCAM dimer. Figure 8b clearly demonstrates the Apt_EpA_ binding configuration of CG_Case1 on EpCAM, and one can see that the Apt_EpA_ fragment from base 3 to 9 closely fits the EpCAM pocket, as indicated by arrows. Consequently, the decrease in EV is much more than that of CG_Case2, and the entropic EV effect for CG_Case1 is more significant, resulting in a significantly lower energy barrier for the Apt_EpA_/EpCAM complex to form.

**Figure 9:**
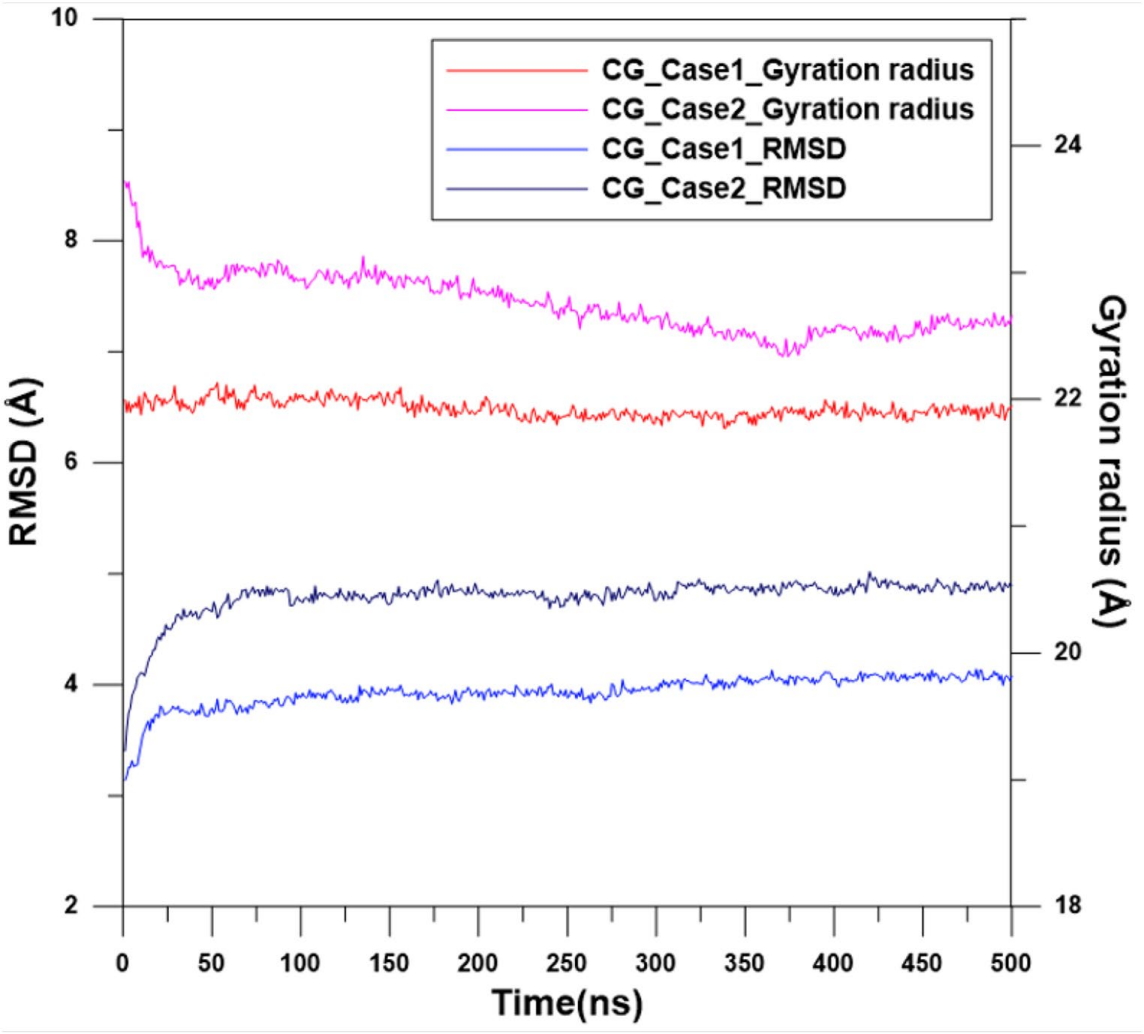
RMSD and gyration radius profiles of the EpCAM in the water environment for CG_Case1 and CG_Case2.

To determine the stability of EpCAMs for CG_Case1 and CG_Case2 at room temperature in the water environment, the CGMD simulation at 300 K was conducted for 500 ns. During this simulation, variations in root mean square deviation (RMSD)^34,51^ and the radius of gyration (Rg)^33^ of EpCAM were used to monitor whether the structure of EpCAM had become stable. The definition of Rg can be expressed by Eq. (4):

4 $$ {\text{R}}_{{\text{g}}} \left( {\text{t}} \right) = \left[ {\sum\limits_{{{\text{i}} = 1}}^{{\text{N}}} {{\text{m}}_{{\text{i}}} \left( {{\text{r}}_{{\text{i}}} \left( {\text{t}} \right) - {\text{r}}_{{{\text{COM}}}} \left( {\text{t}} \right)} \right)^{2} } } \right]^{{\frac{1}{2}}}  $$

where *m*_*i*_ is the mass of the i-th CG bead; *r*_*COM*_(t) is the mass center of the EpCAM at time step *t*; while *r*_*i*_*(t)* represents the coordinates of bead *i* at time step *t*. For any molecule, a larger value of Rg indicates a more expansive structure, and vice versa. In this current study, Rg, however was only used to check the stability of EpCAM rather than how expansive or compact it was. Figure 9 shows the RMSD and Rg profiles of CG_Case1 and CG_Case2 during the MD simulation at 300 K for 500 ns. For CG_Case1, both RMSD and Rg values underwent relatively slight changes for the first 25 ns and then fluctuate around a constant after 25 ns. For CG_Case2, the Rg value reveals that the EpCAM structure requires more time (about 400 ns) to become stable, and its RMSD value is higher than that of CG_Case1, indicating that the configuration of EpCAM in water is more conservative when the Apt_EpA_ binds to EpCAM by attaching to the pocket space of the EpCAM dimer.

**Figure 10:**
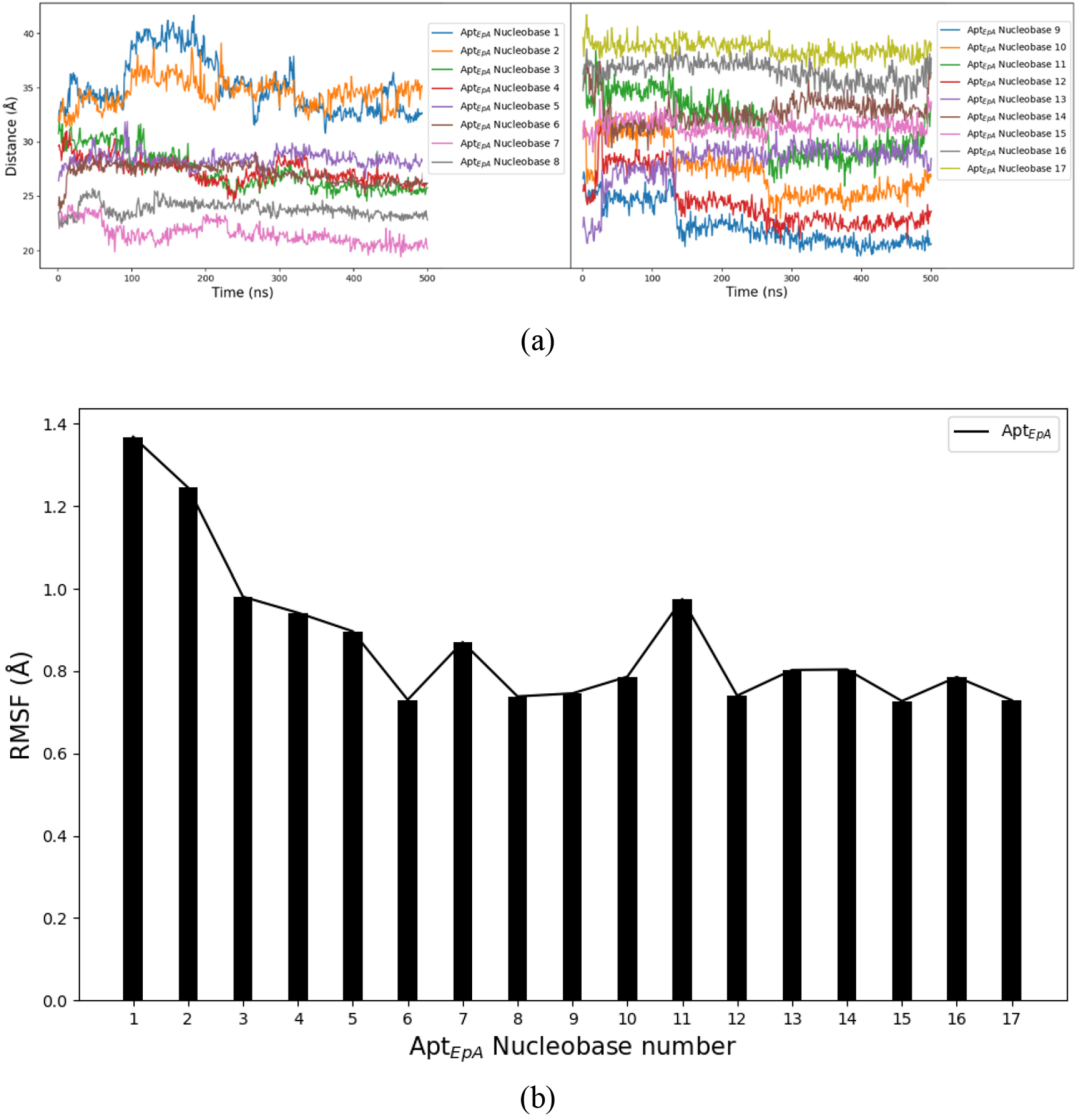
(**a**) Variations of distances between Apt_EpA_ nucleobases and EpCAM mass centre during the CGMD simulation for 500 ns. (**b**) RMSF analysis results for Apt_EpA_ nucleobases.

For investigating the dynamical behaviour of each Apt_EpA_ nucleobase, Fig. 10a shows the variation of distance between respective Apt_EpA_ nucleobase and EpCAM mass centre during the CGMD simulation for 500 ns. Because the Apt_EpA_ backbone beads only undergo the thermal vibration at their adsorption sites of EpCAM, the distance profiles of backbone beads are not shown and discussed. For clearly showing all results, the left and right panels of Fig. 10a demonstrate the distance profiles for Nucleobases 1–8 and Nucleobases 9–17, respectively. During the first 300 ns, the distance profiles of Nucleobases 1, 2, 3, and 4 (shown in the left panel) and Nucleobases 9, 10, 11, and 12 (shown in the right panel) display considerable variations with the simulation time. After 300 ns, one can see all distance profiles fluctuate at constant values, indicating the Apt_EpA_ has reached thermal equilibrium with EpCAM and water beads. The root mean square fluctuation (RMSF) analysis was also used to indicate the flexibility of each Apt_EpA_ nucleobase during the last 100 ns of the CGMD simulation for 500 ns. Figure 10b shows the RMSF result for respective Apt_EpA_ nucleobase and one can see the RMSF values of Nucleobase 1 and Nucleobase 2 are the highest ones, compared to those of the rest 15 Apt_EpA_ nucleobases. Higher RMSF values indicate Nucleobase 1 and Nucleobase 2 display higher flexibility during the CGMD simulation, which also shows the weaker adsorption of this fragment with EpCAM. For the Apt_EpA_ fragment from Nucleobase 3 to Nucleobase 17, the lower RMSF values reveal a stable binding, which contributes the most to the Apt_EpA_ adsorption on EpCAM.

**Figure 11:**
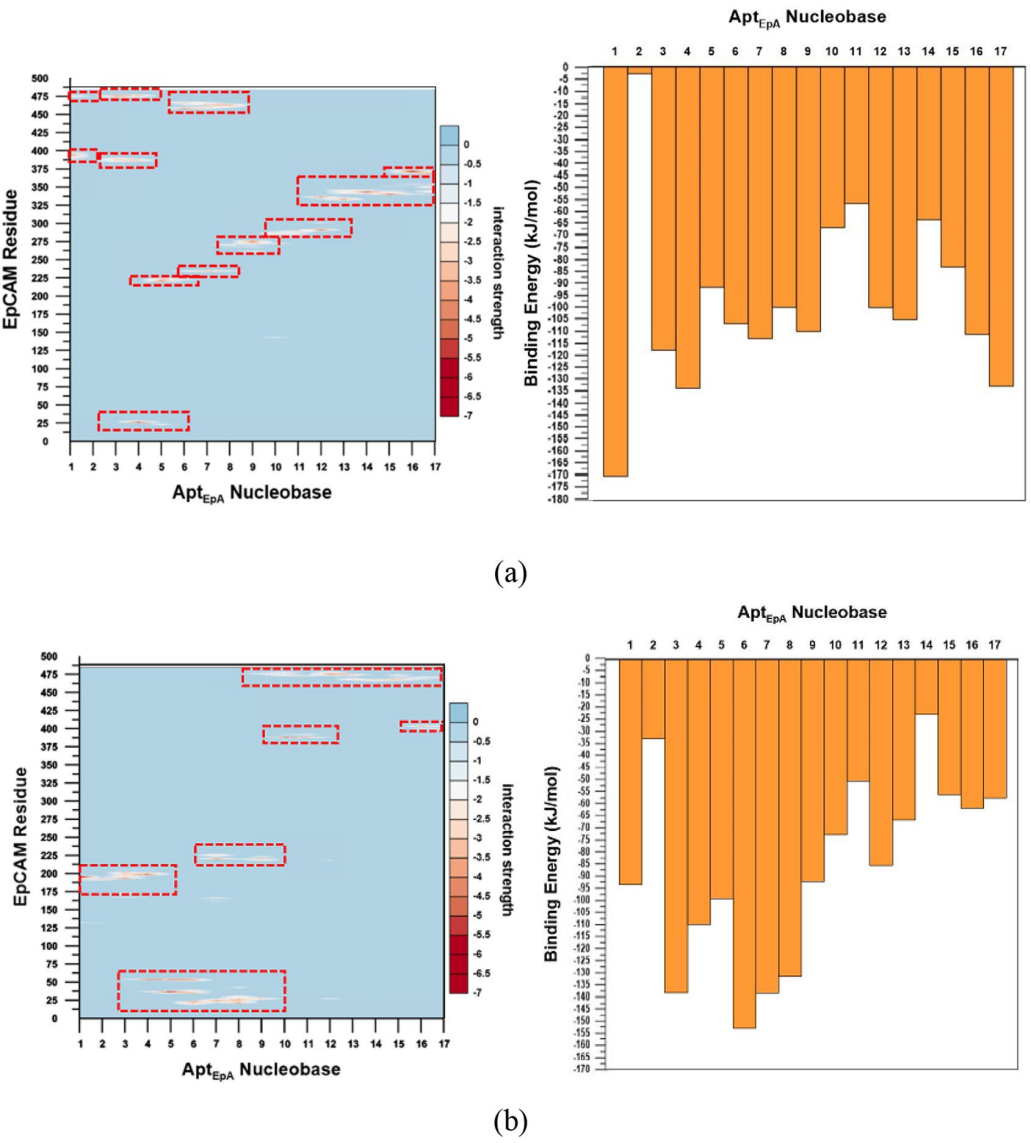
The interaction details between Apt_EpA_ nucleobases and EpCAM for (**a**) CG_Case1 and (**b**) CG_Case2. Left panel: contour map showing the interaction energy distribution of each Apt_EpA_ nucleobase with each EpCAM residue. Right panel: histogram plot showing the interaction energy between EpCAM and an Apt_EpA_ nucleobase in the water environment. The dashed rectangles in the contour maps indicate Apt_EpA_ nucleobases with relatively stronger interaction energies with EpCAM residues.

In order to investigate the interaction strength between each EpCAM residue and each Apt_EpA_ nucleobase, as well as the total interaction strength of each Apt_EpA_ nucleobase with EpCAM, Fig. 11 shows the interaction details between Apt_EpA_ and EpCAM for CG_Case1 and CG_Case2. The contour maps in the left panels show the interaction energy distributions of each Apt_EpA_ nucleobase with each EpCAM residue, while the histogram plots on the right illustrate the interaction energy of each Apt_EpA_ nucleobase with the EpCAM. All interaction energies were sampled by averaging the data from the last 10 ns of CGMD simulation for 500 ns. For the contour maps with the kJ/mol energy scale bar, the vertical axis represents the sequence of the EpCAM amino acid index, while the horizontal axis is the 17 bases of Apt_EpA_ in sequence. For the histogram plots, the vertical axis represents the interaction energy sum of all EpCAM residues to respective Apt_EpA_ nucleobase. For CG_Case1, the interaction energy contour map shows that binding energies lower than − 8.4 kJ/mol widely and continuously span from Apt_EpA_ nucleobases 1–17 around the EpCAM indexes from 225 to 250, 275–300, 325–350, 375–400, and 450–475, as marked by the dashed rectangles. For the interaction energy contour map of CG_Case2, binding energies lower than − 8.4 kJ/mol were also marked by the dashed rectangles, such that it is clear that the distribution of dashed rectangles is relatively more loose and discrete than those in CG_Case1. For the histogram plot of CG_Case2, it can be seen the binding interaction strengths of nucleobases 3–9 are more distinct, while the nucleobase binding interaction strength in CG_Case1 is distributed more uniformly, as can be seen in the histogram plot of Fig. 11a.

## Conclusions

To find the aptamer adsorption configuration on the target molecule more quickly in the water system, the CG model using MARTINI force field is applied for STUN-BH-DMD method. First, the AA model in our previous study is converted to a CG model, and then the MARTINI standard water model is added. Next, the CG model searches directly in the water environment, called CG_Case1, and it was found that the binding energy of CG_AA was 3.1% higher than that of CG_Case1. It also indicates that the STUN-BH-DMD process directly implemented in a water environment can determine the most stable Apt_EpA_/EpCAM complex structure, which matches the experimental environment more closely. We also analyzed the adsorption boundary of these two sets of Apt_EpA_/EpCAM complex structures and found that a small amount of water remained at the adsorption boundary for CG_case1, whereas it could not be found at CG_AA. In CG_case1, these water beads near the Apt_EpA_ terminal also stabilize the interaction between Apt_EpA_ terminals and EpCAM, indicating that water molecules play crucial roles when binding occurs in an aqueous solution.

The nudged elastic band (NEB) method was used to determine the minimum energy pathway (MEP) of Apt_EpA_ adsorption for CG_Case1 and CG_Case2. It is found that the energy barrier of CG_case1 is lower than that of CG_case2, presumably because the adsorption position of CG_case1 is in the pocket-like space of EpCAM. Consequently, the decrease in EV for CG_Case1 is much more than that of CG_Case2 and the entropic EV effect for CG_Case1 is more significant, resulting in a significantly lower energy barrier for Apt_EpA_/EpCAM complex formation.

By monitoring the RMSD and Rg variations during the MD simulation at 300 K for 500 ns in the water environment, it is found that the RMSD and Rg of CG_Case1 reached equilibrium earlier than CG_Case2, and at lower values. From the binding energy contour map and histogram plot of EpCAM and each Apt_EpA_ nucleobase, we can also find that the binding energy of CG_Case1 is more continuous. All these results indicate that CG_Case1 has a better adsorption position than does CG_case2. For CG_Case1, the RMSF analysis result indicates Nucleobase 1 and Nucleobase 2 have higher flexibility during the CGMD simulation, which also shows the weaker adsorption of this fragment with EpCAM. For the Apt_EpA_ fragment from Nucleobase 3 to Nucleobase 17, the lower RMSF values reveal a stable binding, which contributes the most to the Apt_EpA_ adsorption on EpCAM. This study has proposed a new numerical process to find the most stable complex configuration. The CG model reduces computational details and illustrates that a more stable Apt_EpA_/EpCAM complex can be found in water.
